# How do PrP^Sc^ Prions Spread between Host Species, and within Hosts?

**DOI:** 10.3390/pathogens6040060

**Published:** 2017-11-24

**Authors:** Neil A. Mabbott

**Affiliations:** The Roslin Institute & Royal (Dick) School of Veterinary Sciences, University of Edinburgh, Easter Bush, Midlothian EH25 9RG, UK; neil.mabbott@roslin.ed.ac.uk; Tel.: +44-131-651-9100

**Keywords:** prions, prion protein, PrP^Sc^, horizontal transmission, vertical transmission, secondary lymphoid organs, intestine, central nervous system

## Abstract

Prion diseases are sub-acute neurodegenerative diseases that affect humans and some domestic and free-ranging animals. Infectious prion agents are considered to comprise solely of abnormally folded isoforms of the cellular prion protein known as PrP^Sc^. Pathology during prion disease is restricted to the central nervous system where it causes extensive neurodegeneration and ultimately leads to the death of the host. The first half of this review provides a thorough account of our understanding of the various ways in which PrP^Sc^ prions may spread between individuals within a population, both horizontally and vertically. Many natural prion diseases are acquired peripherally, such as by oral exposure, lesions to skin or mucous membranes, and possibly also via the nasal cavity. Following peripheral exposure, some prions accumulate to high levels within the secondary lymphoid organs as they make their journey from the site of infection to the brain, a process termed neuroinvasion. The replication of PrP^Sc^ prions within secondary lymphoid organs is important for their efficient spread to the brain. The second half of this review describes the key tissues, cells and molecules which are involved in the propagation of PrP^Sc^ prions from peripheral sites of exposure (such as the lumen of the intestine) to the brain. This section also considers how additional factors such as inflammation and aging might influence prion disease susceptibility.

## 1. Introduction

The prion diseases are a unique group of sub-acute neurodegenerative diseases that affect humans and certain domestic and free-ranging animals. The infectious prion agent is considered to comprise solely of abnormally folded isoforms of the host-encoded cellular prion protein PrP^C^, termed prion disease-specific PrP^Sc^. Prion infectivity co-purifies with PrP^Sc^ and appears to constitute the major component of the infectious agent [1,2]. The pathology caused during prion disease is considered to be restricted almost entirely to the central nervous system (CNS), where it causes extensive neurodegeneration which ultimately leads to death. The characteristic histopathological features of CNS prion diseases include vacuolation in the brain (spongiform pathology), neurodegeneraton, microgliosis, astrocytosis, and abnormal accumulations of PrP^Sc^. 

Several different forms of prion diseases have been described: spontaneous, genetic, or acquired through various routes of exposure (Table 1). Many prion diseases such as natural sheep scrapie, chronic wasting disease (CWD) in cervid species, and bovine spongiform encephalopathy (BSE) in cattle are considered to be orally-acquired; for example through the consumption of prion-contaminated food or pasture. Examples of vertical prion transmission between an infected mother to the developing fetus or offspring have also been reported. In addition to their important health and economic impacts to livestock industries, some prion disease also have zoonotic potential. The consumption of BSE-contaminated food during the UK BSE epidemic was responsible for the occurrence of a novel human prion disease, variant Creutzfeldt-Jakob (vCJD), which was predominantly described in young adults. Whether other animal prion diseases also have zoonotic potential is an important human health concern. Sporadic CJD (sCJD), in contrast, typically affects elderly individuals and has an unknown etiology and incidence of approximately 1/million population per annum throughout the world. Whether this prion disease is also acquired is uncertain, but a study using experimental mice has proposed that sheep scrapie prions may also have zoonotic potential and cause a disease in the recipients with characteristics identical to sCJD [3]. Instances of accidental iatrogenic prion transmission between humans have also been documented following the transplantation of sCJD-contaminated tissues (dura mater grafts) or tissue products (pituitary-derived human growth hormones), transfusion of blood or blood products from vCJD-infected donors, or use of prion-contaminated surgical instruments or medical devices. 

The first half of this review provides a thorough account of our current understanding of the various ways in which PrP^Sc^ prions may spread between individuals within a population, both horizontally and vertically. Following peripheral exposure, the prions often accumulate to high levels within the secondary lymphoid organs (SLO). This initial replication prion replication phase within the secondary lymphoid tissues is essential for the efficient spread of the prions from the site of exposure to the CNS, a process termed neuroinvasion. The second half of this review goes on to discuss the key tissues, cells, and molecules that facilitate the propagation of PrP^Sc^ prions from peripheral sites of exposure (such as the lumen of the intestine) to the brain. This section also describes how additional factors such as inflammation and aging can influence prion disease susceptibility. 

## 2. Transmission of PrP^Sc^ Prions between Host Species

**Figure 1:**
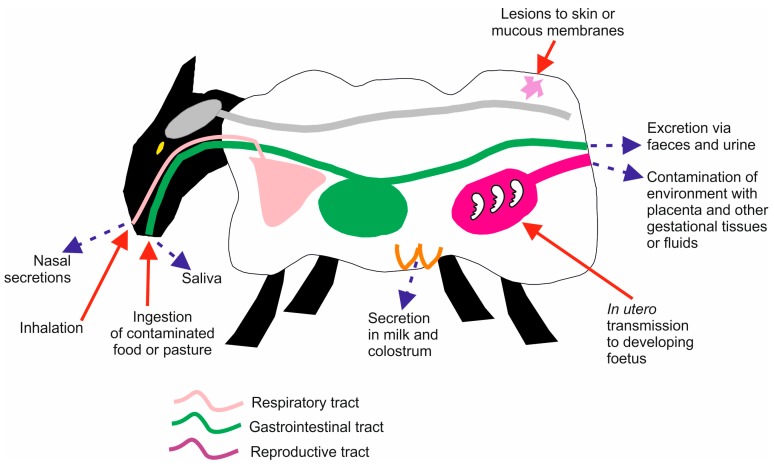
Cartoon summarizing the potential routes of prion exposure in animals such as sheep, and mechanisms in which prions may be disseminated between animals. Red arrows, routes of prion exposure; Broken blue arrows, routes of prion shedding or secretion from an infected animal.

The precise route by which many of the natural prion diseases are acquired or transmitted between host species is uncertain. Prion diseases in animals have the potential to be transmitted by a variety of routes depending on factors such as the stage of host development and the husbandry conditions within which they are maintained. The sections below describe our understanding on contribution of a range of exposure routes summarized in Figure 1 and the factors which can affect disease susceptibility. 

### 2.1. Horizontal Transmission

Many natural prion diseases are horizontally transmitted between host species. Indeed, analysis of mathematical models derived from flocks of sheep affected with natural scrapie, and captive mule deer with CWD, have revealed that horizontal transmission is remarkably efficient and can play an important role in sustaining prion disease epidemics within affected populations [4,5]. The horizontal transmission of CWD between reindeer has also been demonstrated in an experimental setting [6].

#### 2.1.1. Oral/Ingestion

Studies using experimental rodents and domestic animals (sheep, deer and cattle) indicate that the many natural prion diseases such as natural scrapie, CWD, and BSE are most likely to be orally acquired. For example, the oral consumption of meat and bone meal contaminated with BSE prions was responsible for the efficient transmission of BSE prions amongst the UK cattle herd in the late 1980s [7,8]. Indeed, the introduction of control measures to remove ruminant materials from feed was instrumental in controlling the UK cattle BSE epidemic. Unfortunately BSE has since been shown to have zoonotic potential and serious human health concerns, as the consumption of BSE prion contaminated beef products was similarly responsible for the occurrence of vCJD in humans [9,10]. Humans were not the only non-bovine species shown to be susceptible to BSE, as domestic and exotic cats [11] as well as other exotic species such as Arabian oryx, greater kudu, and nyala [12,13] also developed a BSE-related prion disease after consumption of BSE prion-contaminated food. 

#### 2.1.2. Prions Can Be Shed into the Environment and Can Remain Infectious

On the farm and amongst free-ranging animals the horizontal transmission prions most likely occurs via the ingestion of contaminated pasture. Studies of sheep with natural scrapie, cervids with CWD, and experimentally affected rodents show that prion-affected individuals can shed infectious prions into the environment via the excretion of urine [14,15,16,17,18], feces [19,20] and oral (saliva) [15,21,22,23] and nasal [24,25] secretions. Analysis of CWD-infected cervids shows that low levels of prions may be excreted in urine and feces throughout the asymptomatic phase [26]. Furthermore, factors such as inflammation within the kidney (nephritis) may enhance the amount of infectious prions excreted into the urine [27]. The chronic excretion or secretion of prions from affected animals provides the opportunity for significant environmental contamination to occur throughout the disease course. Studies show that on farms with an incidence of prion disease, the prions may persist within the environment for long periods [28]. Prions may persist in soil for at least 18 months [29,30] and can retain their infectious properties, even when bound to plants [31]. Although the mechanism of action is not known, the binding of prions to soil may also enhance their ability to infect the host after oral exposure [32]. The persistence of the prions within the farm environment also introduces practical issues for disease control, as the removal and culling of infected animals from farms on its own may be insufficient to prevent further cases of prion disease occurring when disease-free stock are reintroduced.

#### 2.1.3. Nasal Cavity Is a Potential Portal for Prion Entry

Natural prion disease susceptible animal host species such as sheep, goats and cervids have highly developed olfactory systems which they use to detect food, select mates and sense predators. Although no natural cases of prion transmission via inhalation or the nasal cavity have been reported, experimental studies using mice, hamsters and sheep have shown that prion infection can be established by this route [33,34,35,36,37,38]. These studies imply that small amounts of soil-bound prions might be inhaled and infect the host as the animal forages for food amongst pasture or bedding. 

#### 2.1.4. Lesions to Skin and Mucous Membranes

Intact skin normally acts as a barrier against prion transmission [39], but experimental studies in mice and sheep show that skin lesions also represent an efficient route by which prion infection can be established [39,40]. Lesions to the oral and nasal mucosa similarly enhance prion disease susceptibility, most likely by increasing the efficiency of prion uptake across these epithelial surfaces [41,42,43,44]. 

#### 2.1.5. Accidental Iatrogenic Transmission in Humans

Many instances of accidental iatrogenic CJD transmission have been recorded where disease was transmitted through the use of prion contaminated neurosurgical instruments and stereotactic electroencephalography electrodes, or transplantation of tissues (cornea, dura mater), or preparations (pituitary-derived growth hormone, follicular stimulating hormone) from sCJD-affected cadavers [45]. However, there is no evidence to suggest that these iatrogenic CJD patients are a risk of horizontal sCJD transmission to family members or close contacts [46]. Studies in rodents have also shown that PrP^Sc^ can accumulate in the dental tissues, suggesting the potential for iatrogenic prion transmission during invasive dental procedures such as tooth extractions or root canal treatment [47]. However, no convincing evidence has been found to suggest an increased risk of vCJD transmission due to dental treatment [48].

Data from mice experimentally infected with the mouse-adapted Fukuoka-1 strain of GSS disease have suggested that blood during the clinical phase of disease contains 100 infectious units (IU) of prion infectivity/mL of buffy coat, and approximately 10 IU/mL of plasma [49,50,51]. Much lower levels were detected in buffy coat during the pre-clinical phase, with infectivity undetectable in plasma. A similar level and distribution of infectivity had been shown in mice infected with vCJD [52]. Fractionation of individual blood components from prion infected mice detected low levels of prion infectivity in buffy coat, plasma, cryoprecipitate, and fraction I + II + III [49]. None was detected in association with fractions IV or V [49] or highly purified platelets [53]. 

Two experimental studies in sheep have shown prions can be transmitted to recipients by transfusion of blood from donor sheep which were infected with either natural scrapie or BSE [54,55]. Whole blood or buffy coat drawn during the pre-clinical and clinical phases of disease transmitted disease to at least 10% of the transfusion recipients [54,55]. These studies provided strong evidence that there may be sufficient levels of infectious prions present in the peripheral blood of some pre-clinically affected humans to transmit disease to recipients by transfusion of blood or blood products.

There has been little evidence to suggest that sCJD may be horizontally transmitted between humans by blood or blood products [56]. Several epidemiological case control, look-back, and surveillance studies on sentinel populations such as hemophiliacs have failed to demonstrate an increased risk of sCJD infection due to blood transfusion or exposure to plasma products [56,57,58,59,60]. However a recent study has reported prion infectivity in the plasma of two of four individuals who were infected with sCJD [61]. 

Several vCJD patients are known to have been blood donors. In the UK four cases of vCJD have been reported in recipients of blood or blood products derived from vCJD-infected donors [62,63,64,65]. In the first of these cases [62], a blood donation was made in 1996 and the donor, who was well at the time, went on to develop clinical vCJD, confirmed in 2001. Non-leucodepleted red cell concentrate from this donation was administered to a patient who subsequently developed vCJD. In the second case [63], the donation was made in 1999 and the donor subsequently developed vCJD and succumbed to the disease in 2001. A single unit of non-leucodepleted red cell concentrate derived from this donor was administered to a patient who died of unrelated causes in 2004. Post mortem examination revealed evidence of PrP^Sc^ accumulation only in the patient’s spleen and one of the cervical lymph nodes. To reduce the risk of potential transmission of variant CJD by blood transfusion the UK implemented universal leucodepletion in 1999. This rationale was based on observations that PrP^Sc^ could be detected in lymphoid tissues of vCJD patients [66,67] implying that cells such as lymphocytes might potentially contaminate the blood-stream with prions. Estimates suggest that although leucodepletion can potentially remove approximately 42% of the prion infectivity in blood, a significant fraction remains [68]. This is consistent with the detection of both cell-associated and soluble prion infectivity in peripheral blood [49,50,51]. No new cases of transfusion-associated vCJD have been reported in the UK since 2007 [60]. 

### 2.2. Vertical Transmission

Depending on the host species and prion isolate, infected mothers have been shown to have the potential to transmit prion infection to their offspring. As the examples below show, maternal transmission may play a significant role in sustaining the prion disease prevalence in affected populations. For example, a study of natural scrapie-affected ewes revealed that the incidence of scrapie was increased in their offspring [69]. The risk of developing scrapie was not influenced in the offspring of scrapie-affected sires [69], consistent with the absence of detectable prion infectivity in semen from infected rams [70]. 

Experimental studies using Reeve’s muntjac deer have also demonstrated the potential for CWD to be maternally transmitted in cervid species [71]. This was further supported in a separate postmortem study of maternal and fetal tissues collected from free-ranging Rocky Mountain elk. This study similarly concluded that mother to offspring prion transmission may contribute to the efficient transmission of CWD amongst naturally affected cervids [72]. 

A study of embryos collected from BSE-affected dams suggested that cattle embryos were unlikely to be infected with BSE prions, even when collected from clinically affected mothers, when the risk of maternal transmission may considered the greatest [73]. However, other studies have estimated that the maternal transmission of BSE prions may occur in approximately 10% of calves born to BSE-affected dams [74]. Detailed analyses of UK maternal cohorts has suggested that risk of BSE transmission is increased in calves born to infected dams, especially those born up to two years before the onset of clinical signs of BSE in the dam [75,76]. 

BSE can efficiently transmit to other host species including sheep, goats and mice. Although studies in mice have suggested that maternal BSE prion transmission was possible [74], no evidence of maternal transmission to goat embryos was also reported [77]. If sheep in the UK had been infected with BSE prions during the cattle epidemic, the possibility that maternal transmission might help sustain this disease within the UK sheep flock was an important concern. Maternal BSE transmission in sheep was shown to be possible in an experimental study, but the low frequency at which it occurred was considered to be unlikely to maintain this disease within a population [78]. 

Whether prion disease-affected dams transmit prion disease to their offspring during gestation, or around the time of parturition (birth) has been the subject of much investigation. No cases of scrapie were recorded in offspring derived from the embryos of scrapie-infected dams when they were transferred into scrapie-free recipient sheep [79,80,81]. This implied that embryo transfer could be used as a method to prevent the maternal transmission of natural sheep scrapie, even in offspring with high risk *PRNP* genotypes [82]. These studies also suggested that rather than transmitting disease to the developing fetus in utero, infection from dam to offspring most likely occurred during birth or the post-natal period [83]. 

Placental tissues derived from infected dams may be contaminated with prions [83], and the placenta of goats infected with classical scrapie was able to transmit disease to susceptible goat kids and lambs via oral route [84]. Thus the contamination of pasture or the farm environment with prion-infected placenta or other birth-associated tissues and fluids may contribute to the post-natal transmission of disease between mother and offspring, as well as the horizontal transmission between other animals within the same population [85]. 

However, other studies in scrapie-affected sheep have reported the in utero transmission of prions to the developing fetus [86,87,88,89,90]. The in utero transmission of CWD in free ranging Rocky Mountain elk has also been reported, as PrP^Sc^ was detected in approximately 80% (*n* = 12/15) of the fetuses analyzed from infected dams in one study, regardless of the gestational stage of the fetus [72]. In animals such as sheep where births from multiple fetuses may occur, the sharing of blood components between developing the fetuses in the same uterine horn may aid the dissemination of prions to the cotyledons of fetuses with scrapie-resistant genotypes [91].

The possibility that human prion diseases may be maternally transmitted has obviously raised concern, especially as a number of children have been born to CJD-affected parents. However, current analyses have found no evidence that human prion diseases are maternally transmitted. For example, one study in 2011 analyzed 125 children born to parents who were diagnosed with vCJD [92]. None of these children developed vCJD during the study period or were classified as suffering from a progressive neurodegenerative disorder. The mothers of nine of these children were symptomatic at conception, birth or within a year of clinical onset, and one child was known to have been breast fed. A study in primates also found no evidence of maternal transmission of kuru, sCJD or scrapie [93], consistent with the absence of prion disease-specific PrP^Sc^ in the uterus and gestational tissues, including the placenta and amniotic fluid, of a pregnant woman with sCJD [94]. Of course, obvious caveats to these studies are the small numbers of cases analyzed, and the potentially long duration of the preclinical phase of the disease in the children. However, despite these concerns, the available data do not support the conclusion that human prion diseases can be maternally transmitted. 

#### Milk and Colostrum

The presence of PrP^Sc^ within the mammary glands of scrapie-affected sheep has been reported [95], and the abundance may be enhanced in tissues with chronic inflammation or mastitis [96,97,98]. Colostrum and milk from scrapie-affected ewes have similarly been shown to contain infectious prions [99,100], which may also be enhanced in milk derived from animals with scrapie and mastitis [97]. These studies demonstrate the potential risk of prion spread between sheep and other species through the consumption of sheep milk or milk products. These studies raised important concerns that prions BSE may also be transmitted to humans through consumption of cattle milk or milk products. However, the risk is considered to be extremely low as abnormal PrP was undetectable in the milk from BSE-infected cattle [101]. As mentioned above, no evidence to support a role for vCJD transmission from an infected mother to child in humans has been reported [92]. 

## 3. Transmission of PrP^Sc^ Prions within Host Species

### 3.1. Prions and the Prion Protein

Expression of the cellular prion protein, PrP^C^, is obligatory for cells to be able to replicate prions [102,103]. The prion hypothesis proposes that abnormally folded prion disease-specific PrP^Sc^ proteins are able to self-propagate by recruiting cellular PrP^C^, which is then transformed into the disease-causing PrP^Sc^ isoform [104]. In support of this hypothesis, independent studies have shown that when recombinant mouse PrP is misfolded into the disease-specific form in vitro, the de novo-generated misfolded protein can transmit a prion disease to recipient mice [2,105]. Cellular PrP^C^ is a 30–35 kDa glycoprotein which is encoded by the *PRNP* gene. This protein is expressed on the outer leaflet of the cell membrane via its glycosylphosphatidylinositol (GPI) anchor [106]. The N-terminal portion of the prion protein is mostly unstructured, comprising a long, flexible tail. The secondary structure of the globular C-terminal domain of PrP^C^ contains three α-helices and a short, two-strand β-pleated sheet [107,108]. During prion disease changes occur in the secondary, tertiary and quaternary structures of the PrP molecule, increasing the amount of β-pleated sheet [107,109]. These changes have profound effects on the physico-chemical and biological characteristics of PrP, as the disease-specific PrP^Sc^ isoform is neurotoxic, relatively resistant to proteinase digestion, and accumulates in affected tissues in insoluble aggregates. The precise mechanisms by which these conformational and biological changes occur are unknown, but the requirement for additional chaperone molecules such as RNA, proteoglycans and lipids has been demonstrated [110,111]. Within the CNS it has been revealed that PrP^C^ plays an important role in promoting myelin homeostasis through interactions with the G-protein-coupled receptor Gpr126 (also known as Adgrg6) on Schwann cells [112]. Many other neuronal functions have also been reported, including regulation of circadian rhythms [113], synaptic transmission [114], cognition [115], seizure sensitivity [116], signal transduction [117,118], regulation of apoptosis [119], and protection from oxidative stress [120]. 

Once peripherally acquired PrP^Sc^ prions infect the host via one of the routes described above, many of them first accumulation within the secondary lymphoid organs (SLO) and persist within them at high levels for the duration of the disease. For example, after oral exposure of mice to scrapie prions, the agent accumulates first in the gut-associated lymphoid tissues (GALT) such as the Peyer’s patches [121,122,123]. Similarly, PrP^Sc^ is first detected in the GALT following experimental oral infection of mule deer fawns (*Odocoileus hemionus*) with CWD [124], or sheep with some strains of natural scrapie [125,126,127]. 

PrP^C^ is also expressed in many cell populations within the immune system and SLO, including lymphocytes, leukocytes, granulocytes, mononuclear phagocytes, and stromal cells. Although PrP^C^-deficient mice appear to show no obvious immune deficits [128], PrP^C^ may play a role in cell activation [129,130,131], T cell differentiation [132] and intercellular interactions [133], and phagocytosis [134,135]. The ubiquitous cellular expression of PrP^C^ has important immunological consequences as PrP is tolerated by the host’s immune system. This prevents the development of specific immune responses against PrP^C^ and PrP^Sc^ prions. Thus, the accumulation of prions within SLO does not lead to their eradication from the host, and little evidence of PrP^Sc^/prion-specific immunity has been reported in affected animals [136,137,138].

#### Cellular Sites of PrP^C^ to PrP^Sc^ Conversion

Many studies have attempted to identify the sites of PrP^C^ to PrP^Sc^ (prion) conversion, as such knowledge may reveal novel targets to block the de novo generation of infectious prions within the host. After synthesis, PrP^C^ is first processed in the Golgi before it is expressed upon the plasma membrane [139]. Following subsequent internalization, PrP^C^ traffics to early endosomes. From these the PrP^C^ is sorted either into recycling endosomes and returned to the plasma membrane, or alternatively, sorted into late endosomes for degradation within lysosomes [140]. Several intracellular prion conversion sites have been suggested including the endocytic pathway [141], lysosomes [142], the endosomal recycling compartment [143,144], and the trans-Golgi network [145], implying that PrP^Sc^ traffics along the same endocytic route as PrP^C^. Despite these advances, it is possible that the intracellular location of the prion conversion site varies according to cell type, and host and prion species. For example, high resolution electron microscopy and cryo-electron microscopy studies have not observed any intracellular PrP^Sc^ accumulations within prion-infected stromal follicular dendritic cells in SLO (see FDC below, Section 3.3.6), suggesting that prion conversion in these cell populations occurs upon the cell membrane [146,147]. Cell surface prion conversion may be a more widespread occurrence. Evidence from in vitro studies using neuroblastoma cells has proposed that PrP^Sc^ conversion takes place on the cell within minutes of exposure to prions [148]. The de novo generated PrP^Sc^ is then rapidly endocytosed with some recycled back to the plasma membrane in association with recycling endosome, whereas the remainder predominantly undergoes lysosomal degradation [149]. 

### 3.2. The Accumulation of PrP^Sc^ Prions in SLO is Essential for Their Efficient Spread to the CNS

Studies using immunodeficient mice undertaken across the past four decades have been instrumental in determining the contribution of the host’s SLO and immune cell populations to prion disease pathogenesis. Original experiments using asplenic mice revealed, contrary to expectations, that the early accumulation of prions within the SLO may actually help facilitate efficient CNS infection. These revealed that prion disease survival times after intraperitoneal infection were extended in mice which lacked a spleen [150,151]. Other studies have shown that disease pathogenesis is similarly delayed in the absence of the SLO draining the exposure site, such as the Peyer’s patches in the small intestine after oral prion exposure [122,123,152,153], or the skin draining lymph nodes after infection via skin lesions [154]. 

As well as providing useful biomedical insight into the dissemination and pathogenesis of the acquired PrP^Sc^ prion disease within the host, the information from these studies has proven to have important practical applications. The detection of PrP^Sc^ within the GALT and other SLO soon after exposure has provided a means to identify some prion-infected individuals during the pre-clinical phase [155,156,157,158,159,160]. In a human vCJD-infected patient, PrP^Sc^ was detected within the GALT before the onset of clinical signs [160]. The retrospective analysis of archived appendix and tonsil tissues has since been used in the United Kingdom to provide an estimate of the prevalence of vCJD prions in the human population [161,162,163]. 

### 3.3. The Cellular Dissemination of PrP^Sc^ Prions within the Host

**Figure 2:**
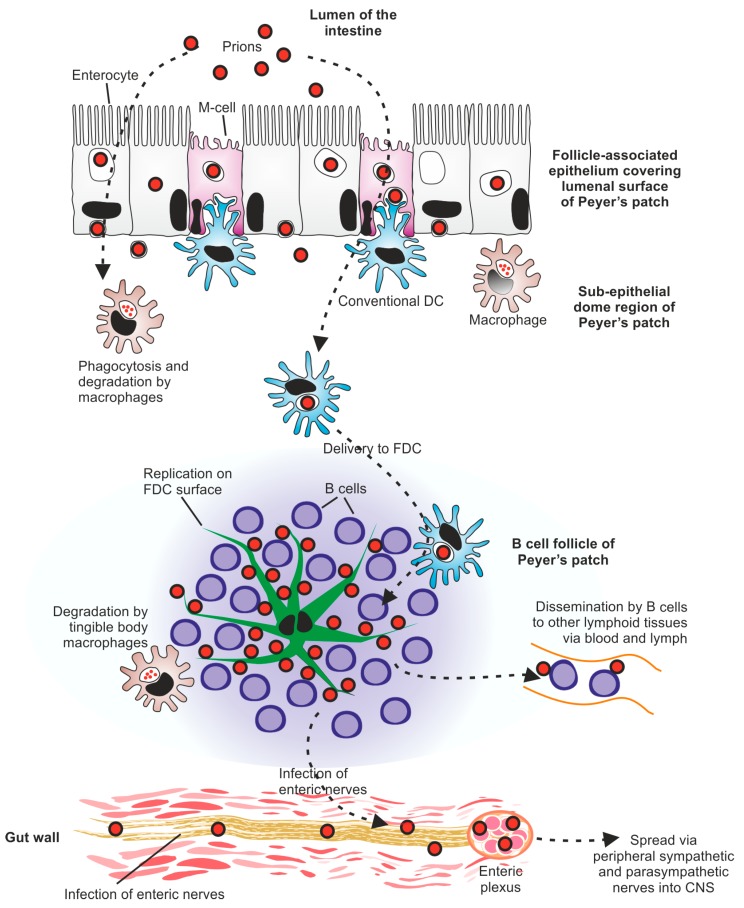
The cells involved in the spread of prions from the intestine to the central nervous system (CNS). After oral exposure the replication of prions upon follicular dendritic cells (FDC) in the Peyer’s patches in the intestine is essential to establish host infection. With the Peyer’s patches, the prions exploit an elegant cellular relay to make their way from the lumen of intestine to the nervous system.

Peripherally-acquired PrP^Sc^ prions appear to exploit an elegant cellular relay to ensure their efficient propagation from the site of exposure to the SLO, where they accumulate before establishing infection within the nervous system (Figure 2). Much of our understanding of this early phase of the disease process has been gained from the study of experimental prion transmissions to a large range of transgenic and immunodeficiency mice, especially those using mouse-passaged scrapie prion isolates. The conclusions from many of these experimental mouse scrapie prions transmissions are discussed below, but comparisons with data from studies of natural prion disease-infected hosts are included where possible. Since many natural prion infections are considered to be orally acquired, these descriptions are mainly focused on the cells and tissues involved in the propagation of prions from the intestine. However, data from other routes of exposure are also discussed at the appropriate places. 

#### 3.3.1. Prions Cross the Gut Epithelium via M Cells

After oral infection, the prions must first cross the gut epithelium, but this single layer of tightly bound epithelial cells acts as an impermeable barrier to macromolecules, the commensal gut microflora, and many orally-acquired pathogenic microorganisms. However, in order for orally-acquired prions to establish infection within the GALT, they first have to cross the gut epithelium in sufficient quantities. The specialized follicle-associated epithelium (FAE) which covers the GALT contains a unique population of highly phagocytic epithelial cells, termed M cells. These cells are specialised for the transcytosis of particulate antigens and microorganisms from the gut lumen into the GALT [164]. The sampling of antigens and pathogens by M cells is important for the initiation of efficient mucosal immune responses against some pathogenic bacteria [165] and the commensal microflora [166]. However, some pathogenic bacteria and viruses have evolved to exploit the transcytotic properties of M cells to cross the gut epithelium and establish host infection [134,167,168,169,170]. 

Prions also appear to exploit M cells to cross the gut epithelium and establish infection with the GALT [171,172,173,174,175]. The accumulation of mouse-passaged ME7 scrapie prions in the GALT and disease susceptibility after oral exposure were both reduced in mice that lacked M cells, or in mice which M cells were transiently depleted before infection [174,175]. Other studies using mouse-passaged RML scrapie prions [171], Fukuoka-1 prions [173], BSE prions [172], and 263 K hamster prions [176] indicate that M cells also play an important role in the transfer of other orally-acquired PrP^Sc^ prion isolates across the gut epithelium. 

Certain pathogenic bacteria [167,177] or inflammatory stimuli such as cholera toxin [178] can increase the density of M cells in the gut epithelium. The differentiation of M cells is dependent on stimulation from underlying stromal cells via their production of the cytokine RANKL [166,179,180]. When mice were treated with RANKL to increase the density of M cells within in the gut epithelium, the uptake of prions from the gut lumen was similarly enhanced, and disease susceptibility was increased by approximately 10-fold [175]. This study indicates that factors that increase the density of M cells in the gut epithelium, such as concurrent pathogen infection, may profoundly affect host susceptibility to orally-acquired prion infections. The binding of CWD prions to soil particles such as smectite clay montmorillonite have also been shown to increase the efficiency of prion uptake from the intestine [30]. Whether the binding of prions to certain types of soil particles enhances their ability to be transferred across the gut epithelium by M cells remains to be determined. 

Data on the role of M cells in the initial uptake of prions from the gut lumen have occasionally been conflicting. Some studies in rodents in which prions were immunohistologically traced after oral exposure have suggested that M cells are the initial sites of prion uptake in the gut epithelium [173,181]. However, other studies in lambs [182] and mice [146] have described M cell-independent uptake pathways. The reasons for these discrepancies are uncertain, and it is plausible that both M cell-dependent and M cell-independent routes may contribute to differing degrees in some circumstances. In vitro-based studies have shown that undifferentiated gut epithelial cell lines (Caco-2 cells) act as a barrier to prion uptake [171], but transcytosis of PrP^Sc^ was evident when it was complexed with ferritin [183]. However, if enterocyte-mediated transfer plays a major role, one would not expect oral prion disease susceptibility to be blocked in mice which specifically lack M cells [174,175]. 

Antigen sampling by M cells has also been demonstrated within the epithelia covering the nasal associated lymphoid tissue (NALT) in the nasal passages [184,185]. After intra-nasal exposure of hamsters to 263 K scrapie prions, transient PrP^Sc^ uptake was detected with NALT-associated M cells. However, a greater abundance of paracellular transport across the epithelia within the nasal cavity was evident [186], indicating that cells involved in the transepithelial transport of PrP^Sc^ prions may vary depending on the exposure route. 

Highly sensitive PrP^Sc^-based detection assays have detected low/trace levels of prions in the blood-stream almost immediately after oral exposure [187,188]. The route through which the prions initially contaminated the blood-stream after oral exposure was not determined, but it was suggested that the amount of PrP^Sc^ that was initially present within the blood-stream was sufficient to establish CNS infection [188]. This conclusion appears to contradict data from many other independent studies which shown that prion replication in the GALT after oral exposure is essential for the subsequent transmission of disease to the CNS [121,122,123,152,153,189]. Thus, although low levels of PrP^Sc^ may be detected in the blood-stream within minutes of oral infection [187,188], the levels within it are insufficient to directly establish infection within the nervous system. 

M cells express a diverse array of receptors on their apical surfaces which specifically bind to certain pathogenic microorganisms [164]. Whether the uptake of prions by M cells is also mediated via by a specific receptor is not known. M cells express PrP^C^ highly [134,190], but PrP^C^-deficiency in the gut epithelium does not affect the uptake of PrP^Sc^ from the intestine [146,173]. 

#### 3.3.2. Conventional Dendritic Cells Aid the Delivery of Prions to SLO

Particles that have been transported across the gut epithelium by M cells are released into the basolateral pocket where they are sampled by mononuclear phagocytes [191]. Mononuclear phagocytes differentiate from bone marrow precursor cells and comprise a heterogeneous population of monocytes, conventional dendritic cells (DC) and tissue macrophages. Conventional DC are strategically positioned to sample their local environment for pathogens and their antigens. After antigen uptake, these cells undergo maturation and migrate towards the draining lymphoid tissue to initiate a specific immune response. Conventional DC possess both degradative and non-degradative antigen uptake pathways to enable them to present processed (partially digested) antigens to T cells or native (intact) antigens to B cells [192,193]. These cells are also centrally involved in the transport of antigens within Peyer’s patches, and on towards the mesenteric lymph nodes [194,195,196]. The migratory characteristics of conventional DC have been exploited by some pathogens to mediate their delivery to SLO [197,198,199,200]. The ability of conventional DC to capture and retain unprocessed antigens [201,202] and migrate into B cell follicles [203,204,205] suggested that classical DC were plausible candidates for the propagation of prions to and within SLO. This hypothesis was supported by the observation some migrating intestinal DC in the afferent mesenteric lymph had acquired PrP^Sc^ after its injection into the gut lumen [206]. Subsequent studies showed that the early replication of prions within the draining SLO was impeded when conventional DC where transiently depleted at the time of exposure [189,207,208,209]. Thus, like certain other pathogens, prions may also exploit conventional DC to establish host infection after peripheral exposure, perhaps using them as “Trojan horses”. 

Whether specific subsets of conventional DC are able to propagate prions to and within SLO is uncertain. However, CD8^+^ conventional DC are unlikely to play a role, as these cells are rarely encountered within the subepithelial dome region immediately beneath the M cell-containing FAE [210], and the specific depletion of CD8^+^CD11c^+^ cells does not influence oral prion disease pathogenesis [208]. Similarly, although prion pathogenesis following infection via skin lesions was impaired in the specific absence of CD11c^+^langerin^−^ dermal DC, the absence of epidermal Langerhan’s cells or langerin^+^ dermal DC had no effect on disease pathogenesis [209]. High levels of infectious prions have also been detected within splenic plasmacytoid DC [211], but these cells are also unlikely to contribute to prion propagation as they do not migrate in the lymphatics [212]. 

Mononuclear phagocytes express cellular PrP^C^ [213,214,215], but the propagation of prions to SLO is not affected by the lack of PrP^C^ expression in hematopoietic cells [216,217,218,219,220,221]. This demonstrates that prions are acquired by conventional DC in a PrP^C^-independent manner, and also that they are not important sites of prion replication. Conventional DC can acquire prions after their opsonization by complement components such as C1q and C3 [221,222]. Depending on their location, phenotype and activation status, conventional DC can express a variety of complement-binding receptors including CR1 (CD35), CR2 (CD21), CR4 (CD11c/CD18), calreticulin, CD93 and SIGN-R1 (CD209b), but whether these also mediate the uptake of prions by conventional DC is uncertain [221,222]. However, prion disease pathogenesis is unaffected in the absence of SIGN-R1 expression at the time of exposure [223]. Conventional DC may simply acquire prions non-specifically by fluid phase micropinocytosis, as they constitutively sample their microenvironments. 

Tunneling nanotubes (TNT) are thin membrane-bound cylinders of cytoplasm which can connect cells to enable cell-to-cell communication and the intercellular transfer of plasma membrane or cytoplasmic components. TNT structures are exploited by HIV-1 as a means of intercellular transfer between T cells [224], and to shuttle virus-encoded immunosuppressive factors from infected macrophages to B cells to suppress host antibody responses [225]. A study has also suggested that intracellular transfer between M cells and neighboring cells can also occur via TNT [226]. In vitro co-culture studies show infectious prions can also transfer between conventional DC and neurones via endolysosomal vesicles within TNT [227,228,229,230]. Whether TNT mediate the intercellular transfer of prions in vivo remains to be determined. Infectious prions can also be released from infected cells in the form of small endosomal-derived vesicles termed exosomes [231] which have the potential to infect neighboring cells [211]. However, the relative contribution of exosomes in this process may vary depending on the prion strain [232]. 

#### 3.3.3. Macrophages Can Phagocytose and Destroy Prions

While some mononuclear phagocytes such as conventional DC can propagate infectious prions to and within SLO, some mononuclear phagocyte populations may sequester and destroy them [233,234]. Tingible body macrophages, for example, are specifically located within the germinal centers of B cell follicles. These macrophages are characteristically loaded with the remnants of phagocytosed apoptotic lymphocytes (tingible bodies), and during prion disease also contain heavy accumulations of PrP^Sc^ within their endosomal compartments [146,147,220]. Transient macrophage depletion prior to peripheral prion exposure has been shown to enhance the accumulation of PrP^Sc^ within SLO [235,236]. These data suggest that macrophages typically scavenge and degrade prions in an attempt to protect the host from infection. 

The burden of infectious prions within the SLO rapidly reaches a plateau level within a few weeks of exposure which is maintained for the duration of the infection [216,237]. How this plateau is maintained is uncertain. It is plausible that a competitive state is reached within these tissues whereby the rate of prion amplification matches the rate of degradation by macrophages [234,235]. 

#### 3.3.4. Cell Free

Although data from many of the studies described suggest that prions are propagated to and within SLO in a cell-associated manner, the possibility that a fraction of the prions are also conveyed in a cell-free manner cannot also be excluded [40,186,209,221].

#### 3.3.5. B Cells Indirectly Support Prion Replication in SLO

In stark contrast to their susceptibility to infection with most other pathogenic microorganisms, mice which lack mature B and T cells, such as severe combined immunodeficient (SCID) mice, *Rag*-1^−/−^, *Rag*-2^−/−^, and *Agr*^−/−^ mice [238,239,240,241], are refractory to peripheral prion infection. However, the T cells themselves do not influence prion disease pathogenesis as prion accumulation in SLO and neuroinvasion are not affected in T cell deficient thymectomised mice [151,242], or in transgenic mice with specific T-cell deficiencies (CD4^−/−^, CD8^−/−^, *β*2-*µ*^−/−^, TCRα^−/−^ or *Perforin*^−/−^ mice) [217,240]. T cells appear to lack the cellular factors required to sustain prion infection as even the artificial ectopic expression of high levels of PrP^C^ in T cells (20 × *Prnp* copies) is insufficient to sustain prion infection within them [243]. 

In the SLO of some prion-infected hosts, heavy accumulations of disease-specific PrP are detectable within the B cell follicles [124,125,160,238,244]. In the specific absence of B cells, the accumulation of prions in the spleen and subsequent neuroinvasion are both significantly reduced [240]. However, B cells also do not replicate prions as transgenic mice which express high levels of PrP^C^ only on B cells were also unable to directly replicate prions [245]. This indicated that B cells most likely played an indirect role in prion pathogenesis, perhaps through the provision of homeostatic support to other cell populations. 

#### 3.3.6. Follicular Dendritic Cells Retain and Replicate Prions

SCID mice and other B cell-deficient mouse lines are also indirectly deficient in follicular dendritic cells (FDC), as these cells require constitutive stimulation from B cells to maintain them in their differentiated state [246,247,248,249]. FDC are an important stromal cell subset that resides within the B cell follicles and germinal centers of SLO. FDC differentiate from ubiquitous perivascular precursor cells (pericytes) and are a distinct lineage from the bone-marrow-derived classical DC described above (Section 3.3.2) [250,251,252]. Immunohistochemical analysis shows that prions accumulate upon FDC in the SLO of experimentally-infected mice, some sheep with natural scrapie, cervids with CWD and patients with vCJD [124,125,160,238,244]. As discussed below, the accumulation and replication of certain prion strains upon FDC is essential to establish host infection and neuroinvasion. 

The transfusion of SCID mice with wild-type (immunocompetent) bone marrow restores the B and T cells in these mice, and by doing so, indirectly induces the maturation of FDC in their SLO [246]. Coincident with the induction of FDC maturation, this treatment also renders the mice susceptible to peripheral prion infection [216,217,218,241]. 

B cells express the cytokines tumor necrosis factor (TNF)α and lymphotoxins (LT), which are the essential stimuli that maintain FDC differentiation [248]. In the absence of these cytokines, the FDC rapidly de-differentiate [248,253,254,255,256,257]. Prion accumulation in SLO and neuroinvasion are significantly impaired in mice deficient in TNFα or LT stimulation, demonstrating the requirement for FDC in the establishment of prion infections [123,237,258]. Prion accumulation in SLO and disease susceptibility are also reduced when the FDC are temporarily de-differentiated by administration of soluble receptors which block the LT- or TNFα-mediated signaling between the B cells and FDC [121,259,260,261,262,263]. Although FDC are important for maintaining germinal center responses, an absence of germinal centers or germinal center B cells alone does not influence prion disease pathogenesis or susceptibility [237,264]. 

FDC characteristically trap and retain native antigens on their cell surfaces, which they display to B cells within the follicle and germinal center. The long-term retention of these antigens by FDC helps to promote immunoglobulin (antibody)-isotype class switching, affinity maturation of naïve B cells and the maintenance of immunological memory [265,266,267,268,269,270,271]. By secreting the factor MFGE-8 (which specifically binds to phosphatidylserine on the surface of apoptotic cells) the FDC also mediate the phagocytosis of apoptotic B cells by tingible body macrophages [272]. The ability of FDC to retain native antigens for long periods raised the possibility that they might also simply trap and retain prions produced by other infected cell populations such as neurones. Several studies have exploited the non-haematopoietic-origin of FDC [246,250] to help address this issue. Mismatches were created in *Prnp* gene expression between the FDC-containing stromal and lymphocyte/leukocyte-containing compartments of the SLO by transfusing hematopoietic cells from PrP^C^-deficient mice into PrP^C^-expressing (wild-type) mice, and vice versa [216,217,218]. In these studies, the FDC were derived from the recipient, whereas all the hematopoietic cell populations were derived from the donor bone marrow. When these mice were infected with prions, prion accumulation upon FDC was only detected in the spleens of mice which had a PrP^C^-expressing stromal compartment. These studies provided strong evidence that FDC were important sites of prion accumulation in SLO. However, roles for other stromal cells could not be entirely excluded as in these studies it was not possible to dissociate the PrP^C^ expression status of the FDC from that of the nervous system, or other stromal cell populations within the SLO. 

FDC and mature B cells express high levels of *Cr2* which encodes the complement receptors (CR) CR2/CR1 (CD21/35) [273,274]. CD21-cre mice [273] have been used to specifically control *Prnp* expression only in FDC, enabling PrP^C^ expression to be “switched on” or “switched off” only in FDC [220]. The expression of PrP^C^ only on FDC was sufficient on its own to sustain high levels of prion replication in the spleen. Conversely, prion replication in the spleen was blocked when PrP^C^ expression was specifically ablated only on the FDC. Data from all the above studies together definitively show that FDC are the essential sites of prion replication in SLO. 

After their replication upon FDC within the SLO, the prions subsequently infect both the sympathetic and parasympathetic nervous systems and spread along the nerves within them to the CNS, where they ultimately cause neurodegeneration [176,275,276]. The role of FDC in prion disease pathogenesis appears to be to amplify the prions above the threshold required for neuroinvasion. How the prions subsequently infect the peripheral nervous system is uncertain, as no significant direct physical contacts or synapses between FDC and nerves has been described. However, the rate of neuroinvasion from SLO is influenced by the distance between FDC and the peripheral sympathetic nerves [262]. 

#### 3.3.7. FDC Acquire Prions as Complement-Opsonized Complexes

FDC express high levels of PrP^C^ [216,217,220,277], but many other cell lineages also express PrP^C^ highly but do not play an essential role in prion disease pathogenesis. Clearly, other FDC characteristics in addition to PrP^C^ expression enable them to replicate prions. FDC have many slender dendritic processes which extend throughout the B cell follicle. These dendrites enable the FDC to trap and retain large amounts of native antigen upon their surfaces in the form of immune complexes, consisting of antigen-antibody and/or opsonizing complement components. Complement components C1q and the regulatory protein factor H can bind to PrP^Sc^ [278,279], and the specific absence of opsonizing complement components (C1q, C2, C3, C4 and factor H) or CR expression on FDC impedes prion accumulation in the spleen and delays neuroinvasion [274,280,281,282,283,284]. Comparison of the relative contributions of the CR1 and CR2 receptors has revealed a more prominent role for CR2 in prion disease pathogenesis [285]. Activation of the terminal complement activation pathway leads to formation of the membrane attack complex which can lyse the target cells. Deficiency in complement component C5 (an important component of the membrane attack complex) in contrast, has no influence on prion disease pathogenesis [286]. This suggests that soon after the prions infect the host, they are bound by soluble complement components and are acquired by FDC in the SLO as complement-opsonized complexes in a CR-dependent manner [274,280,281,282,286]. 

The immune complexes that are retained by FDC are initially internalized before undergoing cyclical rounds of display on the FDC surface [287]. This cyclical mode of immune complex expression on the FDC surface helps to protect the antigens from degradation, enabling them to be retained for much longer periods [271]. Despite this, high resolution immunohistochemical analyses indicate that prion replication occurs on the FDC surface, as PrP^Sc^ has not been detected within them [146,147,288,289,290]. This appears to be in contrast to prion infection in nerves where PrP^Sc^ conversion has been demonstrated within the endosomal recycling compartment [143]. 

#### 3.3.8. Conventional DC Can Shuttle Prions towards FDC

Chemokines help to attract lymphocytes and leukocytes to SLO and control their positioning within them. The chemokine CXCL13 is expressed by FDC and other stromal cells in the B cell-follicles of lymphoid tissues and recruits CXCR5-expressing cells towards them [291,292]. The migration of certain populations of conventional DC towards the FDC-containing B cell-follicles is also mediated by CXCL13-CXCR5 signaling [203,205,293]. In the specific absence of CXCR5-expressing conventional DC, the early accumulation of prions upon FDC in Peyer’s patches was impeded [294]. This suggests that once the prions have been transported across the gut epithelium by M cells, they are subsequently acquired by conventional DC [146,189] and propagated by them in a CXCL13-CXCR5-dependent manner towards the FDC within the B cell-follicles of Peyer’s patches [294]. The prions are then acquired by FDC and amplified upon their surfaces above the threshold required to achieve neuroinvasion [121,123,146,153,220]. 

The positioning of conventional DC within the inter-follicular T-cell regions of the Peyer’s patches, and their steady-state migration from Peyer’s patches to the mesenteric lymph nodes are both dependent upon CCR7-CCL19/CCL21-signaling [295]. Consistent with the demonstrations that T cells do not influence prion disease pathogenesis [240], an absence of CCR7-CCL19/CCL21-signaling does not influence oral prion disease pathogenesis [296]. 

Although the transient depletion of CD11c^+^ cells or deficiency in CXCR5-expressing conventional DC impedes the accumulation of prions in Peyer’s patches and reduces disease susceptibility, a small number of mice in these studies did develop clinical prion disease [189,294]. This indicates that conventional DC provide an efficient route by which prions are initially conveyed to FDC. However, in the absence of conventional DC at the time of oral exposure, small quantities of prions are able to avoid clearance by cells such as tissue macrophages [235,236], and establish infection upon FDC via less efficient routes [40,209,221]. 

Once the prions have been transported towards the B cell follicles by conventional DC, it remains to be determined how they are subsequently transferred to the FDC. Follicular B cells in the sub-capsular sinus region of the lymph nodes can acquire lymph-borne immune complexes via their CR and deliver them to FDC [297,298,299]. In the spleen, marginal zone B cells play a similar role in the shuttling of blood stream-borne immune complexes to FDC [300]. Once the B cells are within the follicle, the higher immune complex-binding affinities of the FDC enables them to strip the immune-complexes from the surfaces of the B cells. Conventional DC can retain PrP^Sc^ on both the cell surface and in intracellular compartments [221]. The higher expression levels of CR on the cell membranes of FDC may similarly enable them to strip complement-opsonized prions from conventional DC.

#### 3.3.9. FDC-Independent Prion Accumulation and Neuroinvasion

Although many prion isolates in different host species may replicate first upon FDC in the SLO, examples have been described where prion accumulation in SLO and/or neuroinvasion occur independently of FDC. The factors which determine the requirement for FDC in prion disease pathogenesis are uncertain, but this may be influenced by prion agent strain, host species, *PRNP* genotype, and exposure route. The dose of prions used to infect the host can also influence disease susceptibility, as high doses can bypass the requirement for amplification within SLO prior to neuroinvasion [258]. The infecting prion strain and host *PRNP* genotype can influence disease pathogenesis in sheep [125,301]. Some prion diseases, such as sporadic CJD in humans are not associated with early prion accumulation in the periphery tissues including the SLO [302], and the susceptibility mice to the mouse-adapted FU agent strain of CJD prions was unaffected by an absence of mature B cells and FDC [303]. BSE prions in cattle are considered to have little SLO involvement during the preclinical phase [304], but PrP^Sc^ and/or infectious prions have been detected in the small intestines of most cattle after experimental (oral) infection and some cattle after natural exposure [305,306,307]. However, when BSE prions are transmitted to other species such as humans (in the form of vCJD), sheep and mice, their accumulation in the lymphoid tissues is a characteristic feature [66,308,309]. Whereas mouse-adapted ME7 prions are unable to accumulate in the spleens of TNFα-deficient mice due to an absence of FDC [237], this is not true for RML prions, which can also accumulate within the high endothelial venules of lymph nodes [258,310]. Inflammatory stromal cells that are a distinct lineage from FDC have also been shown to have the potential to replicate prions under certain circumstances [311]. 

Despite the presence of FDC within the NALT, prion neuroinvasion after exposure via the nasal cavity can occur independently of the NALT and SLO, implying direct infection of the nervous system [35,37].

### 3.4. Prion Infections Cause Limited Pathology in SLO

The accumulation of PrP^Sc^ within the CNS ultimately leads to the development of neuropathology. Despite the detection of high levels PrP^Sc^ upon FDC in the SLO throughout the duration of the disease, no gross immunological deficiencies have been reported [312,313,314,315]. However, ultrastructural analysis of prion-affected SLO has revealed evidence of morphological changes to the FDC. These include adversely affected maturation cycles, abnormal dendritic folding and exacerbated accumulation of immune complexes between the FDC dendrites [147,289]. The immunological consequences of these pathological disturbances to FDC and germinal centers are uncertain, as antibody production appears to be unaffected in prion-infected animals [312]. 

### 3.5. Orally-Acquired Prions Replicate First in the GALT of the upper Gastrointestinal Tract

The FDC within the Peyer’s patches in the small intestine are the essential early sites of prion replication and neuroinvasion in mice, as oral prion disease susceptibility is blocked in their absence [122,123,152,153,175]. Many natural prion diseases also accumulate upon FDC in the large intestinal GALT after oral exposure, such as the recto-anal mucosa-associated lymphoid tissues (RAMALT) of scrapie-affected sheep and goats, and CWD-affected deer and elk [155,156,157,158,316,317,318]. The detection of PrP^Sc^ within these tissues has helped to detect prion-infected animal and individuals during the pre-clinical phase [155,156,157,158,159,160], and has been used in the UK to estimate the prevalence of vCJD in the human population [161,162,163]. However, studies using mice have shown that the large intestinal GALT are not important early sites of prion accumulation or neuroinvasion from the intestine [153].

Detailed analyses of sheep with natural scrapie has revealed a similar mode of pathogenesis. Scrapie prions are first detected in the GALT of the upper gastrointestinal tract before spreading to the draining lymph nodes and onwards to other lymphoid tissues. The large intestinal GALT, such as the caecal patches, were not early sites of prion accumulation [319,320,321,322]. A similar temporal distribution was observed after oral exposure of sheep to BSE [323] and in humans with vCJD, where the distribution of PrP^Sc^ in lymphoid tissues is restricted during the pre-clinical phase, and more wide-spread at the clinical stage [63,67]. Likewise, RAMALT biopsy studies in scrapie-infected goats [316] and CWD-infected white-tailed deer [317,324] show lower incidences of prion accumulation in the RAMALT during the earlier stages of disease, supporting the notion that these tissues are not early sites of prion accumulation. Indeed, an analysis of CWD prevalence in elk showed that PrP^Sc^ was not reliably detected in the RAMALT until it was also detectable in the CNS [318], consistent with the conclusion that the large intestinal GALT become infected with prions until much later in the disease process [157,325]. Experimental transmissions of CWD to white-tailed deer have identified the oropharynx as the initial site of prion entry after oronasal exposure [326]. The possibility therefore cannot be excluded that biopsy specimens of large intestinal GALT may miss individuals if sampled during the early stages of oral prion infection, and significantly underestimate the disease prevalence. 

Antigens from the intestinal lumen may be delivered directly to the mesenteric lymph nodes [327,328]. Prions are similarly detected upon FDC in the mesenteric lymph nodes and the spleen soon after oral exposure (most likely after their dissemination from the Peyer’s patches). The absence of the mesenteric lymph nodes does not affect prion neuroinvasion or disease susceptibility [123,153,175]. This implies that the levels of prions initially delivered to the mesenteric lymph nodes immediately after oral exposure are insufficient to establish accumulation and replication upon the FDC within them. Consistent with the conclusion that the GALT of the upper gastrointestinal tract are the essential sites of prion accumulation and neuroinvasion after oral prion exposure, the absence of the spleen also does not influence oral prion disease pathogenesis or susceptibility in mice [294,329].

### 3.6. B Cells Aid the Spread of Prions between SLO

After residing within the Peyer’s patches, B cells migrate to the mesenteric lymph nodes and then return to the circulation [330]. B cells can recirculate between lymphoid tissues for several weeks [331] and often acquire antigens from FDC as they migrate through the germinal centers within them [332]. Studies in mice have suggested that recirculating B cells also appear to mediate the initial propagation of prions from the draining lymphoid tissue to other SLO. When the migration of B cells between SLO was specifically blocked, the dissemination of prions from the draining SLO to other SLO was also blocked [333]. Whether the same occurs in natural prion infections is uncertain, but prions have been detected in association with lymphocytes [334,335] and B cells in the blood of sheep with scrapie [336] and deer with CWD [337]. 

### 3.7. Prion Infection of the CNS Occurs via the Sympathetic and Parasympathetic Nervous Systems

The circumventricular organs in the bran are sites of molecular exchange between the blood-stream and the CNS. The detection of prion disease-specific PrP within these brain regions has been described in some scrapie-affected sheep, suggesting the potential for the hematogenous spread of prions into the CNS [338]. However, monocytic infiltration into the circumventricular organs is not observed in prion disease-affected hosts, arguing against the cell-associated hematogenous spread of prions into the CNS. Furthermore, studies in mice show that an absence of recruitment of circulating monocytes into the CNS does not influence prion disease pathogenesis within the CNS [339]. Although infectious prions are present in the blood-stream of vCJD-infected individuals, the spatial distribution of the PrP^Sc^ deposits in the brain in relation to the blood vessels also does not support a major role for the heematogenous spread of vCJD prions into the CNS [340]. 

The heematogenous spread of prions directly into the CNS cannot be entirely excluded, as low/trace levels of PrP^Sc^ may be initially detected with the CNS soon after exposure, using highly sensitive assays [188]. However, data from many studies suggests that the prions initially establish infection within the CNS after their spread from the SLO along peripheral nerves. The SLO and the GALT are highly innervated with sympathetic neurones [341]. Detailed immunohistochemical tracing studies show that after replication upon FDC in the GALT, orally acquired prions subsequently infect the enteric nervous system and spread along sympathetic (e.g., splanchnic nerve) and parasympathetic (e.g., vagus nerve) efferent nerves fibers to infect the CNS [146,176,275,342,343,344,345]. Furthermore, the specific depletion of sympathetic nerves, impairs prion neuroinvasion from SLO [276]. Conversely, prion disease pathogenesis is exacerbated in mice in which the density of sympathetic nerves in SLO is increased [276], or in those in which the distance between FDC and sympathetic nerves is reduced [262]. 

Within the intestine, mononuclear phagocytes are abundant in the muscular layer where they interact with enteric nerves to regulate gastrointestinal motility [346,347]. Data from in vivo and in vitro studies have proposed that prion-infected conventional DC or other mononuclear phagocytes may also play a role in the transfer prions to peripheral nerves [228,229,230,348]. 

### 3.8. Effect of Inflammation and Pathogen Co-Infection on Prion Disease Pathogenesis

The majority of the studies above describe prion disease pathogenesis during the steady state. However, inflammation can have a significant impact on prion disease pathogenesis, either by enhancing the uptake of prions from the exposure site, or by expanding their tissue distribution. For example, pathology to the gut mucosa such as that caused by bacterial colitis [349], enhanced M cell-density [175], or lesions to the mucosal surfaces of the oral [43] or nasal [44] cavities can each enhance disease susceptibility by increasing the prion uptake. Mitogen stimulation or repetitive immunization to non-PrP antigens around the time of peripheral prion exposure can also similarly increase disease susceptibility [350,351]. Chronic inflammation, by inducing the formation of FDC-containing ectopic tertiary lymphoid tissues, can expand the distribution of prions within the infected host [27,123,153,352,353]. The effects of chronic inflammation on prion disease pathogenesis could have important human and animal health consequences and aid their vertical and horizontal transmission, for example, by enhancing the burden of prions secreted into milk (in animals with mastitis) or urine (in animals with nephritis). 

Despite the apparent widespread exposure of the UK human population to BSE prions during the BSE epidemic (approximately 500,000 infected cattle are estimated to have entered the food chain [354]) the numbers of clinical cases of vCJD in humans have fortunately been rare (Reference [355]; 178 definite or probable cases, as of 2 October 2017; www.cjd.ed.ac.uk). This does however, raise the possibility that oral prion susceptibility may differ between individuals. Studies using transgenic mice which expressed human PrP^C^ proposed that a significant species barrier restricts BSE transmission to humans [356]. After interspecies prion exposure, the processing and amplification of prions upon FDC in SLO is important for their adaptation to the new host, and to achieve neuroinvasion [309,357]. How this host adaptation occurs is not known, but may be influenced by the sialylation status of the PrP^Sc^ [358]. It is plausible that inflammation [175] or enteritis [349] may enable a greater burden of prions to be acquired from the gut lumen, increasing the probability that more will be able to avoid clearance by cells such as macrophages [123,235]. This may help to reduce the transmission barrier to some orally acquired prion strains by providing a greater opportunity for the prion quasi-species with zoonotic potential to be selected and to undergo adaptation and amplification upon FDC [282]. 

### 3.9. Effects of Host Age on Prion Disease Pathogenesis and Susceptibility

Clinical sporadic CJD cases have predominantly occurred in the elderly (median age at onset of disease = 67 years), whereas the majority of the clinical vCJD cases have almost exclusively occurred in young adults (median age at onset of disease = 26 years). The age-related incidence of vCJD is not simply due to the exposure of young adults to greater levels of BSE prions through dietary preference [359]. Aging has a profound effect on immune function, termed immunosenescence [360,361,362]. As a consequence of this immunosenescence, the elderly respond less effectively to vaccination, have increased susceptibility to viral and bacterial infections and increased incidence of cancer and autoimmune diseases. Immunosenescence can also influence the pathogenesis of peripherally acquired prion infections, by impairing prion accumulation/replication in SLO and reducing disease susceptibility. In sheep, cattle, cervids, and humans, susceptibility to peripheral prion infections is associated with GALT development [363]. For example, in Cheviot sheep, a marked fall in the size of their ileal Peyer’s patches and lymphoid follicle density is apparent from the onset of puberty [364]. 

In aged (≥600 days old) mice the development and function of FDC [365] and M cells [366] which play a central role in prion neuroinvasion is adversely affected. The expression of PrP^C^ by aged FDC is also reduced [365]. In the spleen, the marginal zone forms a barrier around the lymphocyte-containing white pulp, and is important for the capture of blood-borne antigens and immune complexes. The immune complexes are then captured by marginal zone B cells and delivered to the FDC in the B cell follicles [300,367]. Due to gross disturbances to the splenic marginal zone, the FDC in aged mice are also unable to efficiently trap immune complexes and prions on their surfaces [277,309,368] (see author’s online video https://media.ed.ac.uk/media/1_4wmqldeh). These ageing-related impairments to M cells and FDC impede the accumulation of prions in the SLO of aged mice, reducing disease susceptibility [277,309,365].

Although peripherally-exposed aged mice did not develop clinical prion disease, PrP^Sc^ was detected in some of their brains but was undetectable in their spleens [365]. This has important implications for the reliability of preclinical diagnostic tests based on the detection of PrP^Sc^ in blood or SLO, as these may be much less sensitive when used on elderly individuals. For example, low numbers of lymphoid follicles were shown to be present in RAMALT biopsy specimens from older elk (*Cervus elaphus nelson*; >8.5 years old) [369] and mule deer (*Odocoileus hemionus*; ≥3 years old) [370] reducing the reliability of the histopathological detection of PrP^Sc^ within the RAMALT of CWD-affected animals. 

In pre-weaning animals, the developmental status of the GALT or intestine can also have a profound influence oral prion disease susceptibility. As a consequence of the underdeveloped status of FDC in neonatal mice, prion accumulation in their SLO after peripheral exposure is reduced and neuroinvasion is delayed [371,372]. The GALT tissues are more developed in neonatal sheep when compared to mice. However, the oral susceptibility of lambs to BSE prions is greater during the pre-weaning stage than during the post-weaning stage [373]. Multiple factors are likely to contribute to this age-related difference in susceptibility, but differences in gut development, mucosal permeability, and possibly also the presence of maternal immunoglobulin may each enhance the transfer of PrP^Sc^ across the gut epithelium [374]. 

## 4. Opportunities for Prophylactic and Therapeutic Intervention

At the time of writing, no safe or effective treatments had been developed for clinical use to block or prevent further spread of prion diseases in humans or domestic animals. However, many varied approaches have been reported. Unfortunately, some potentially useful anti-prion drugs identified from in vitro studies have had quite differing efficacies when translated in vivo into animal models or prion disease-affected patients. 

### 4.1. PrP^Sc^ as a Therapeutic Target

A large variety of experimental in vitro studies have attempted to identify potential drugs or compounds which may block prion conversion or accumulation within cells. From these, many have been translated into animal models, and a small number have made it into clinical trials in human prion disease patients. 

#### 4.1.1. Quinacrine

Quinacrine is an antimalarial drug which can also inhibit prion accumulation in infected cells [375]. However, subsequent studies showed this drug had no effect on survival times in mice experimentally infected with prions [376]. Two independent clinical studies in humans also failed to demonstrate any beneficial effect of quinacrine treatment on the clinical course of prion disease in affected patients [377,378]. 

#### 4.1.2. Pentosan Polysulphate

Pentosan polysulphate is a polyanion and heparin analogue which can significantly extend survival times, or reduce the susceptibility of mice to peripherally administered prions when administered around the time of infection [379], or by direct intraventricular infusion into the brain [380]. This compound has also been administered to some human CJD patients in the UK and Japan. In these limited trials, pentosan polysulphate was administered continuously to the brain by intraventricular infusion. Treatment may have extended survival times in some patients, but no apparent improvement in clinical signs were reported [381]. 

#### 4.1.3. Tetracyclic Antibiotics

The tetracyclic antibiotics doxycycline and tetracycline can reduce prion infectivity and inhibit the neurotoxicity of PrP-peptides in vitro [382,383]. Unfortunately, the results of clinical trials using doxycycline in human patients with clinical prion disease were negative [384]. 

### 4.2. Prions as Anti-Prions

A series of studies undertaken in the 1970’s showed that the infection of mice with a prion agent strain with a long disease duration could block subsequent infection with a prion agent strain with a shorter disease duration [385,386]. This competition between prion agents raised the hypothesis of whether such blocking could be achieved against prion diseases in domestic animals or humans through administration of prion agent strains with incubation periods which exceed the longevity of the host, or the use of synthetic molecules. Later independent studies showed that an attenuated “slow” strain of mouse-passaged CJD prions could similarly impede the pathogenesis of subsequent a infection with a more virulent or “faster” prion agent strain [387]. On a similar theme, a recent novel approach generated non-pathogenic, self-replicating PrP^Sc^-like forms, termed “anti-prions”. A single injection with these apparently innocuous PrP^Sc^-like forms into hamsters infected with 263 K prions was shown to be sufficient to compete with prion replication and delay survival times [388]. Substantial safety trials and risk assessments will need to be undertaken before the efficacy of such approaches are tested in domestic animal species and human prion disease patients. 

### 4.3. Targeting Prion-Induced Neurodegeneration

Detailed analyses of the molecular mechanisms by which prions cause neurodegeneration have begun to identify potential molecular targets for intervention during prion disease. For example, prion replication in the brain leads to sustained over-activity of the unfolded protein response that controls the initiation of protein synthesis. This causes a persistent repression of protein translation which ultimately leads to synaptic failure and neuronal death [389]. Oral treatment of mice with a specific inhibitor of the kinase PERK (protein kinase RNA-like endoplasmic reticulum kinase), a key mediator of the unfolded protein response pathway, can prevent the development of neurodegeneration and clinical prion disease [390]. 

### 4.4. Immunization

There have been many elegant experimental attempts to develop novel immunotherapeutic approaches to induce host immunity to prions [391,392]. A major barrier to the effectiveness of many of these is T cell-tolerance towards PrP due to the widespread expression of cellular PrP^C^ throughout the mammalian body. This has made the development of effective PrP^Sc^ prion-specific vaccines extremely challenging. However, studies predominantly performed in mice have shown that immunization against PrP or the passive administration of PrP-specific monoclonal antibodies are potential approaches to block prion disease transmission [393,394]. At the time of writing, the MRC Prion Unit in the UK planned to undergo a clinical trial to passively administer a small number of sCJD patients with a human anti-PrP antibody to attempt to block further neurodegeneration. 

Mucosal vaccination appears to be the most appropriate method for prophylactic protection against orally acquired prion infections [395,396]. However, a mucosal vaccine may offer little protection against accidental iatrogenic CJD transmissions where infection occurs via intravenous transfusion of contaminated blood or blood products, transplantation of tissues, or use of contaminated surgical instruments. Therefore, a useful anti-prion vaccine should be able to induce both strong mucosal and systemic anti-PrP antibody responses. 

Since the cellular prion protein is almost ubiquitously expressed, the potential for an anti-prion vaccine to recognize host PrP^C^ and cause autoimmunity must not be ignored. However, detailed comparisons of several anti-prion antibodies have identified those that target the α1 and α3 helices of PrP^C^ can induce rapid neurotoxicity [397].

## 5. Conclusions

Substantial progress have been made in our understanding of how PrP^Sc^ prions disseminate between and within individuals. However, many important issues with implications for animal and human health remain unresolved. The transmission of prions from one host species to other hosts of the same species is typically efficient, and causes disease in the recipients with highly reproducible disease characteristics. However, inter-species prion transmissions upon first passage are typically characterized by their low efficiency and extended disease durations. This effect on prion transmission is termed the ‘species barrier’ effect. Many factors are known to have an important influence on the inter-species transmission of prions such as polymorphisms and mutations in the *PRNP* gene, and biophysical aspects of templating events are clearly important (see above). Unfortunately the precise molecular mechanism/s responsible for the species barrier effect is uncertain. An ability to predict the potential for a novel prion isolate to have the potential to transmit to other species, especially humans, is crucial to restrict and control future prion disease outbreaks. 

Despite the widespread exposure of the UK population to BSE-contaminated food in the 1980s, there have fortunately been much fewer human clinical vCJD cases than anticipated. However, retrospective analyses of human tonsils and appendix indicate a higher incidence of pre-clinically infected individuals (~1/2000) than the clinical data suggest [161,162,163]. This implies that many individuals may harbor detectable levels of prions in their tissues in the absence of clinical signs, and the potential existence of a subclinical carrier state. The factors which can influence the progression of CNS prion disease in preclinically-affected individuals are not known. A thorough understanding the factors which can influence the progression of the preclinical phase will identify those which enhance the risk of developing clinical prion disease.

Once the prions have been amplified on the surfaces of FDC above the threshold required for neuroinvasion, they infect the enteric nerves within the intestine [146,176,276]. Although the relative positioning of the FDC and sympathetic nerves within the SLO appears to influence the rate of neuroinvasion [262], but how the infection is propagated between FDC and enteric nerves is unknown. By identifying how the prions initially infect the nervous system may identify a novel methods to block the spread of prions to the CNS. 

Unfortunately there are no safe or effective treatments which can be used to cure or block further spread of these devastating neurodegenerative diseases in humans. Some exciting experimental advances have been made, but trials in larger animal species including humans will be required to determine their efficacy and safety. Currently, advances in gene editing techniques are rapidly enhancing the ability to repair the genomes of human cell populations to treat certain previously incurable diseases associated with specific gene mutations. Whether a similar gene-editing approach can be used to block prion infection in host tissues by inserting protective mutations within the *PRNP* gene [398] is an exciting prospect for future research. 

## Figures and Tables

**Table 1:** PrP^Sc^ prion diseases of humans and animals.

Prion Disease	Affected Species	Transmission Route
Iatrogenic Creutzfeldt-Jakob disease (CJD)	Human	Accidental medical exposure to CJD-contaminated tissues or tissue products
Sporadic Creutzfeldt-Jakob disease	Human	Unknown. Theories include somatic mutation or spontaneous conversion of PrP^c^ to PrP^Sc^
Variant Creutzfeldt-Jakob disease	Human	Ingestion of BSE-contaminated food or transfusion of blood or blood products from variant CJD-infected blood donor
Familial Creutzfeldt-Jakob disease	Human	Germ-line mutations of the *PRNP* gene
Gerstmann-Straussler-Scheinker syndrome	Human	Germ-line mutations of the *PRNP* gene
Kuru	Human	Ritualistic cannibalism
Fatal familial insomnia	Human	Germ-line mutations of the *PRNP* gene
Bovine spongiform encephalopathy	Cattle	Ingestion of contaminated food
Scrapie	Sheep, goats, mouflon	Acquired. Ingestion, horizontal transmission, vertical transmission unclear
Chronic wasting disease	Elk, deer, moose	Acquired, ingestion, horizontal transmission, vertical transmission unclear
Transmissible mink encephalopathy	Mink	Acquired (ingestion) source unknown
Feline spongiform encephalopathy	Domestic and zoological cats	Ingestion of BSE-contaminated food
Exotic ungulate encephalopathy	Nyala, kudu	Ingestion of BSE-contaminated food
